# Supplementary material for: Lysosomal TBK1 Responds to Amino Acid Availability to Relieve Rab7-Dependent mTORC1 Inhibition
Source: bioRxiv. 2023 Dec 17:2023.12.16.571979. Preprint. [Version 1] doi: 10.1101/2023.12.16.571979 (PMC10760094; doi:10.1101/2023.12.16.571979)
Supplement: Supplement 1 [file media-1.pdf]

Supplemental Data for:

Lysosomal TBK1 Responds to Amino Acid Availability to Relieve Rab7-Dependent mTORC1 Inhibition

Gabriel Talaia[1,2,3,4,6], Amanda Bentley-DeSousa[1,2,3,4,6] and Shawn M. Ferguson[1,2,3,4,5,6*]

Departments of Cell Biology[1] and Neuroscience[2], Program in Cellular Neuroscience, Neurodegeneration and Repair[3], Wu Tsai Institute[4], Kavli Institute for Neuroscience[5], Yale University School of Medicine, New Haven, Connecticut 06510, USA. Aligning Science Across Parkinson's (ASAP) Collaborative Research Network, Chevy Chase, MD, 20815, USA.[6]

*Correspondence: shawn.ferguson@yale.edu

Supplemental Figures

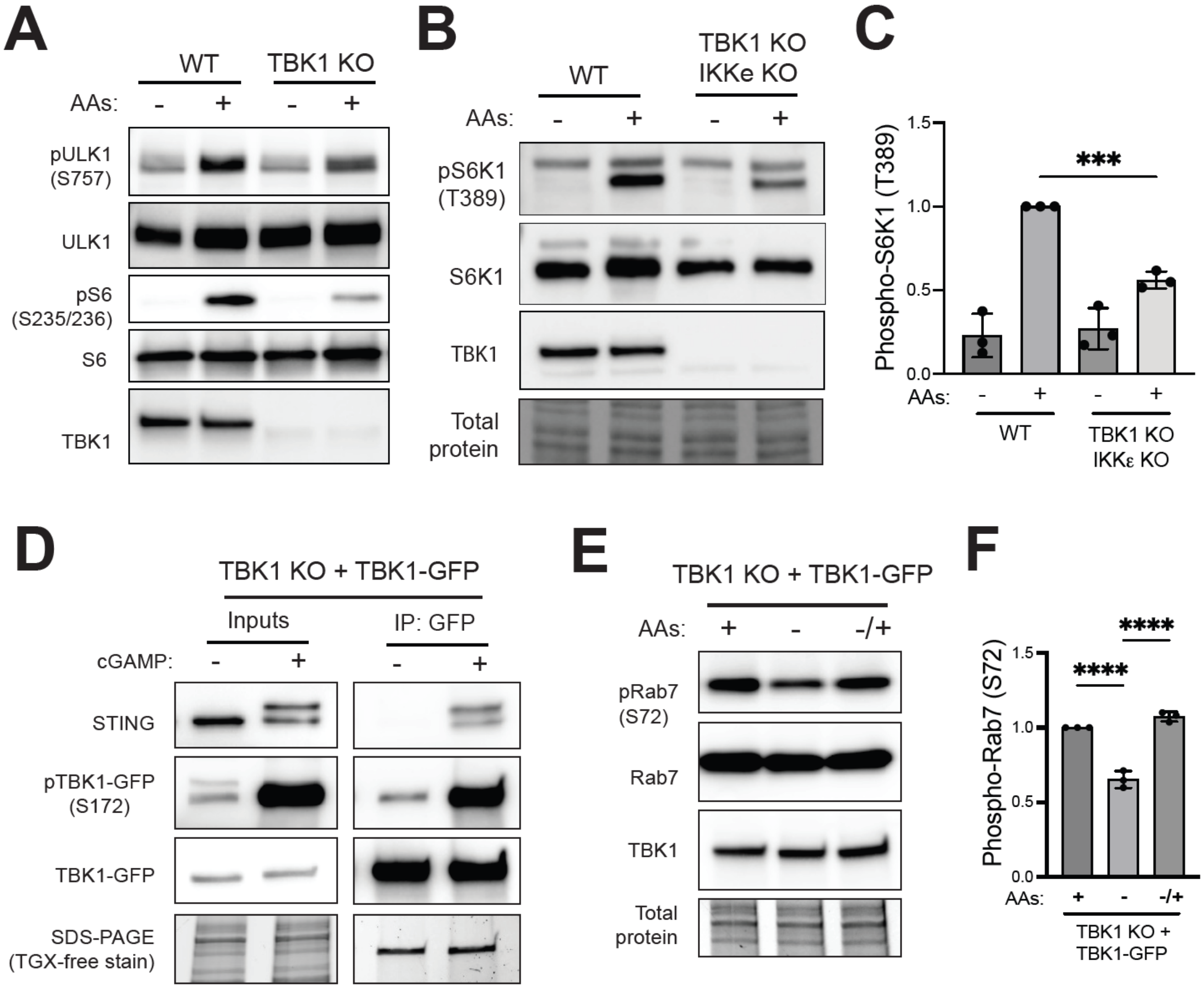

Figure S1: TBK1 is required for efficient amino acid dependent mTORC1 and cGAMP dependent STING activation. (A) Immunoblot analysis of phospho-ULK1 at S757, total ULK1, phospho-S6 (S235/S236) and TBK1 of WT and TBK1 KO HeLa cells starved for 60 min (-) and re-fed with amino acids (+). (B) Immunoblot analysis of S6K1 phosphorylated at Thr389 [pS6K1 (T389)], total S6K1 and TBK1 in whole-cell lysates from WT versus TBK1 KO RAW 246.7 cells that were starved of amino acids for 60 minutes (-) and then re-fed with amino acids for 60 minutes (+). TGX stain-free method was used to visualize total protein. (C) Quantification of phospho-S6K1 (T389) normalized to total S6K1 and expressed as a fold change

compared to the WT cells under re-fed conditions. Statistical significance was determined by ordinary one-way analysis of variance (ANOVA) with Šidák *post hoc* test (n=3; mean ± SD; ***, $p<0.001$). (D) Immunoblot analysis of STING, phospho-TBK1-GFP and total TBK1-GFP in the lysates (Inputs) and immunoprecipitated TBK1-GFP (IP: GFP) of TBK1 KO HeLa cells rescued with TBK1-GFP untreated (-) or treated with cGAMP (70 µM) for 120 min (+). (E) Immunoblot analysis of pRab7 (S72), total Rab7 in cell lysates of TBK1 KO + TBK1-GFP HeLa cells under basal fed conditions (+), starved (-) and amino acid re-fed (-/+). (F) Phospho-Rab7 levels were quantified and normalized to total Rab7 (basal conditions were considered 1). Statistical significance was determined by ordinary one-way ANOVA with Šidák post-test (n=3; mean ± SD; ****, $p<0.0001$).

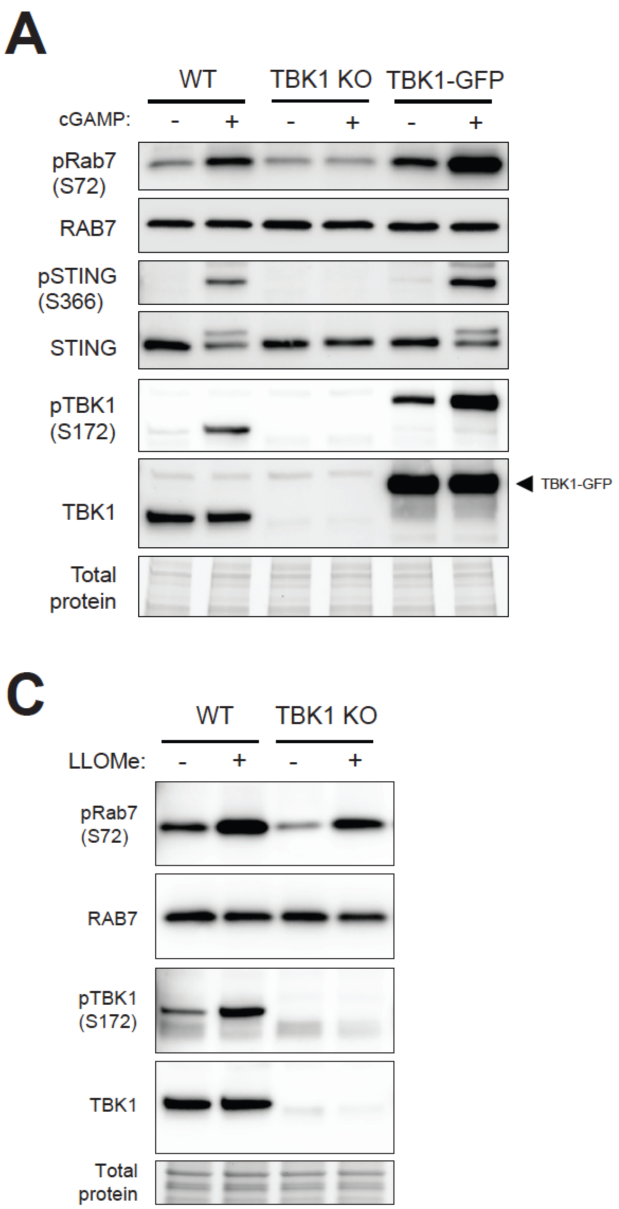

Figure S2: TBK1 activity is regulated by multiple signals. (A) Immunoblot analysis of the indicated proteins in WT, TBK1 KO and TBK1-GFP HeLa cells untreated (-) or treated with cGAMP (70 μM) for 120 min. (B) Immunoblot analysis of the indicated proteins of WT and STING KO RAW 246.7 cells starved for 60 min (-) and then refed with amino acids for 60’ (+). (C) Immunoblot analysis of the indicated proteins in WT and TBK1 KO HeLa cells untreated (-), DMSO 0.1 % (v/v), or treated with LLOMe (1 mM) for 30 min.

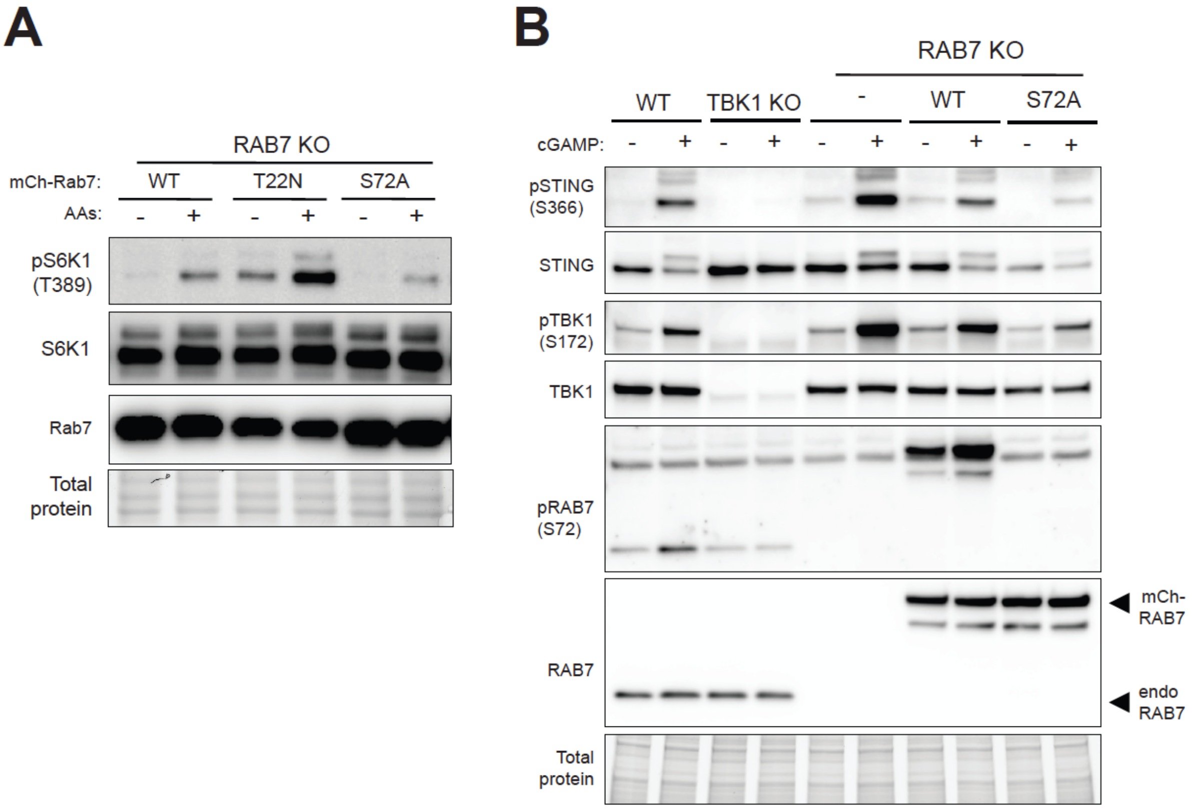

Figure S3: Rab7 mutants show defective mTORC1 and STING signaling. (A) Immunoblot analysis of the indicated proteins of Rab7 KO HeLa cells transiently transfected with mCherry-tagged wild-type Rab7 and mutants (T22N and S72A), starved for 60 min (-) and then refed with amino acids for 60 min (+). (B) Immunoblot analysis of the indicated proteins of WT, TBK1 KO, Rab7 KO and Rab7 KO stably expressing mCherry-tagged wild-type or S72A versions of Rab7 HeLa cells untreated (-) or treated with cGAMP (70 μM) for 120 min.

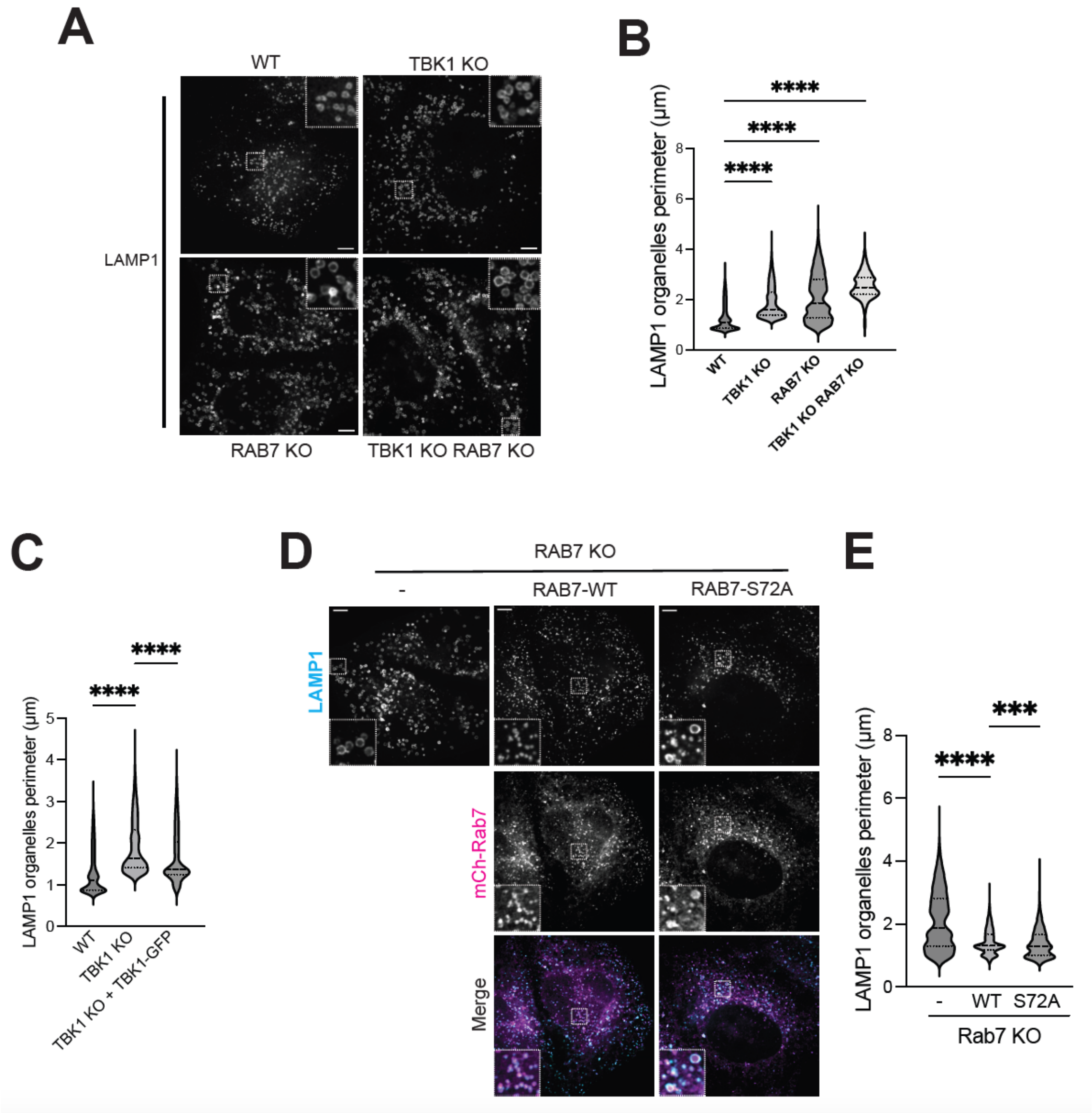

Figure S4: TBK1 regulates lysosome size independent of Rab7. (A) Immunofluorescence microscopy analysis of LAMP1 in basal conditions of WT, TBK1 KO, RAB7 KO, TBK1 KO RAB7 KO HeLa cells. (B) Quantification of of LAMP1-positive organelle (lysosomes) perimeter (µm) in cells of the indicated genotypes. Scale bar: 5 µm. (C) Quantification of LAMP1-positive organelle (lysosomes) perimeter from WT, TBK1 KO and TBK1-GFP rescue data presented in Figure 1G. (D) Immunofluorescence microscopy analysis of LAMP1 and mCh-Rab7 in basal conditions for Rab7 KO HeLa cells versus Rab7 KO stably expressing RAB7-WT or RAB7-S72A. (E) Quantification of LAMP1-positive organelle (lysosomes) perimeter (µm) in cells of the indicated

genotypes. Data plotted in panels B, C and E represents results from 2-4 independent experiments; 11-29 regions of interest; 1-3 cells per region of interest. Statistical significance was determined by the Kruskal–Wallis's test followed by Dunn's post test (Violin Plot; ****, $p<0.0001$; ***, $p<0.001$). Scale bar: 5 µm.

Supplemental Methods

Supplemental Methods

Table 1 - Summary of cell lines used in this study.

Cell Line	Genotype	Reference
HeLa M	WT	Pietro De Camilli, Yale University
HeLa M	TBK1 KO	This work
HeLa M	TBK1 KO + TBK1-GFP	This work
HeLa M	TBK1 KO + TBK1-E696K-GFP	This work
HeLa M	RAB7 KO	This work
HeLa M	RAB7 KO + mCherry-RAB7	This work
HeLa M	RAB7 KO + mCherry-RAB7-S72A	This work
HeLa M	TBK1 KO + RAB7 KO	This work
RAW 264.7	WT	ATCC
RAW 264.7	STING KO	Bentley-DeSousa and Ferguson, 2023
RAW 264.7	TBK1 KO + IKK KO	Bentley-DeSousa and Ferguson, 2023

Table 2 - Summary of nutrients and drug treatments used in this study

Compounds/Drugs	Company	Product Number
DMEM	Thermo Fisher Scientific	11965-092
HI-FBS	Thermo Fisher Scientific	16140-071
Penicillin/Streptomycin (10,000 U/mL)	Thermo Fisher Scientific	15140122
Puromycin	Thermo Fisher Scientific	A11138-03
RPMI 1640 Medium Modified w/o Amino acids	USBiological	R9010-01
MEM Amino Acids	Gibco	11130-051
2',3'-cGAMP	Chemietek	CT-CGMAP
BX-795	Cayman Chemical	14932
LLOMe	Cayman Chemical	16008

Table 3 - Summary of plasmids used in this study

Plasmid	Reference
pSpCas9-2A-Puro-TBK1-gRNA (PX459)	This paper
pSpCas9-2A-Puro-RAB7A-gRNA1 (PX459)	This paper
pSpCas9-2A-Puro-RAB7A-gRNA2 (PX459)	This paper
pEIF1A-piggyBac transposase	Michael Ward (NINDS) (Pantazis et al., 2022)
pPB-EF1A-Puro-hTBK1-EGFP	This paper
pPB-EF1A-Puro-hTBK1-E696K-EGFP	This paper
pPB-EF1A-Puro-mCherry-RAB7	This paper
pPB-EF1A-Puro-mCherry-RAB7-S72A	This paper
pCMV-mCherry-RAB7	Addgene #61804
pCMV-mCherry-RAB7-S72A	This paper

pCMV-mCherry-RAB7-T22N	This paper
pCMV-myc-RAB7	Christopher Burd, Yale University
pCMV-myc-RAB7-T22N	Christopher Burd, Yale University

Table 4 - Sequences of oligonucleotide primers used in this study

**Primer**	**Sequence (5'-3')**
RAB7_F	CAAAAAAGCAGGCTGCCACCATGGTGAGCAAGGGCGAG
RAB7_R	TCAGCAACTGCAGCTTTCTG
PB-EF1_F	CAGAAAGCTGCAGTTGCTGAACCCAGCTTTCTTGTACAAAGTG
PB-EF1_R	GGTGGCAGCCTGCTTTTTTG
RAB7-S72A_F	ACGGTTCCAGGCTCTCGGTGT
RAB7-S72A_R	TCCTGTCCTGCTGTGTCC
RAB7-S72E_F	ACGGTTCCAGGAGCTCGGTGTGG
RAB7-S72E_R	TCCTGTCCTGCTGTGTCC
TBK1-E699K_F	ATTAAAGGAAAAGATGGAAGG
TBK1-E699K_R	TTCTTCATACCAAGAGTC
TBK1 gRNA sense	CACCGCATAAGCTTCCTTCGTCCAG
TBK1 gRNA antisense	AAACCTGGACGAAGGAAGCTTATGC
RAB7A gRNA1 sense	CACCGGTCATCCACCATCACCTCCT
RAB7A gRNA1 antisense	AAACAGGAGGTGATGGTGGATGACC
RAB7A gRNA2 sense	CACCGCATTCAAAACCCTAGATAGC
RAB7A gRNA2 antisense	AAACGCTATCTAGGGTTTTGAATGC

Table 5 - Description of antibodies used in this study

**Antibody**	**Company**	**Concentration**
S6K1	Cell Signaling Technologies	1:2000
P-S6K1 (T389)	Cell Signaling Technologies	1:1000
ULK1	Cell Signaling Technologies	1:2000
P-ULK1 (S757)	Cell Signaling Technologies	1:2000
S6	Cell Signaling Technologies	1:6000
P-S6 (S235/236)	Cell Signaling Technologies	1:2000
mTOR	Cell Signaling Technologies	1:2000
Rab7 (E9O7E)	Cell Signaling Technologies	1:4000
P-Rab7 (S72)	Abcam	1:1000
STING	Cell Signaling Technologies	1:1000
P-STING (S366)	Cell Signaling Technologies	1:1000
TBK1	Cell Signaling Technologies	1:2000, 1:500
P-TBK1 (S172)	Cell Signaling Technologies	1:1000, 1:100
LAMP1 (D2D11)	Cell Signaling Technologies	1:2000
LAMP1 (H4A3)	DSHB	1:2000
GFP	Invitrogen	1:500

PDI	Cell Signaling Technologies	1:1000
GM130	BD Biosciences	1:2000
Rabbit IgG (HRP)	Cell Signaling Technologies	1:2000
Mouse IgG (HRP)	Cell Signaling Technologies	1:2000
Biotin (HRP)	Cell Signaling Technologies	1:4000
Alexa 488-Rabbit	Invitrogen	1:600
Alexa 568-Rabbit	Invitrogen	1:600
Alexs 647-Mouse	Invitrogen	1:600
